# Post-Traumatic Stress Disorder, Mobile Phone Dependence, and Academic Boredom in Adolescents During the COVID-19 Pandemic

**DOI:** 10.3389/fpsyg.2021.724732

**Published:** 2021-11-01

**Authors:** Lingyan Shen, Xinyue Wu, Rui Zhen, Xiao Zhou

**Affiliations:** ^1^Jing Hengyi School of Education, Hangzhou Normal University, Hangzhou, China; ^2^Department of Psychology and Behavioral Sciences, Zhejiang University, Hangzhou, China

**Keywords:** PTSD, mobile phone dependence, academic boredom, adolescent, COVID-19

## Abstract

**Background:** The coronavirus disease (COVID-19) pandemic has threatened adolescents’ mental health and even elicited their academic problems. Post-traumatic stress disorder (PTSD) is one of the most common negative psychological reactions, and academic boredom is a typical academic problem to the pandemic. PTSD might be related to academic boredom, but the underlying mechanism of this potential relation in the context of the COVID-19 pandemic remains unclear.

**Aims:** Under the framework of the job demands–resources model and the model of compensatory internet use, this study aims to examine the mediating role of mobile phone dependency in the relation between PTSD and academic boredom.

**Methods:** Six hundred and thirty-one middle school students in Hubei Province were investigated using self-report questionnaires. SPSS19.0 and Mplus7.0 were used for data analysis.

**Results:** PTSD symptoms were associated positively with academic boredom, and mobile phone dependence played a mediating role in the relation between PTSD and academic boredom. Specifically, adolescents with severe PTSD symptoms tended to report greater dependency on mobile phones, and hence show higher levels of boredom in learning.

**Conclusion:** PTSD symptoms of adolescents directly aggravated their academic boredom, and indirectly affected academic boredom by increasing their dependence on mobile phones.

## Introduction

At the end of 2019, the coronavirus disease (COVID-19) pandemic spread rapidly across the world, and it has had various negative effects on adolescents’ mental health (Marchini et al., 2020; Wiguna et al., 2020; Zhang et al., 2020). Post-traumatic stress disorder (PTSD) is one of the most common negative psychological reactions to the pandemic (Imran et al., 2020; Yue et al., 2020; Zhen and Zhou, 2021). PTSD not only leads to cognitive and emotional problems (Dekel et al., 2013; Xu et al., 2017), but also causes academic difficulties in adolescents (Zhou et al., 2017b, 2018). Thus, the relation between PTSD and academic difficulties should be concerned in the context of the COVID-19 pandemic.

Since the outbreak of the COVID-19 pandemic, there have been frequent reports of adolescents who are tired of studying, which has aroused widespread concern. Academic boredom refers to a negative attitude toward learning, during which students experience emotional exhaustion (Lin and Huang, 2014; Ying et al., 2016; Supervía et al., 2020). This can directly lead to a decline in academic performance (May et al., 2015), which in turn reduces students’ academic involvement, damages their interpersonal relationships with classmates and teachers, and affects students’ long-term career development (Perry et al., 2010; Rojas-Flores et al., 2015; Quin, 2016). Therefore, it is particularly important to investigate the academic boredom of adolescents during the COVID-19 pandemic.

According to the job demands–resources model (Schaufeli and Bakker, 2004), PTSD symptoms may trigger a sense of academic boredom in adolescents. This model claims that an individual’s work condition depends on two main factors: job resources and job demands. Job resources provide support and assistance to individuals, and job demands are the continuous physical and mental efforts invested. Insufficient resources and high demands may cause job burnout (Cadime et al., 2016; Qi and Wu, 2018). Adolescents with PTSD symptoms already have a high level of psychological stress, accompanied by related behavioral and physiological problems, such as negative cognition (Xu et al., 2017), insomnia, and hyperarousal (Zhou et al., 2014). In the process of coping with these problems, adolescents may suffer from a lack of physical and mental resources. Engaging in learning activities (such as attending lectures and doing homework) requires adequate physical and mental resources; students with PTSD symptoms might lack the physical and mental resources required to meet academic demands, thus resulting in a sense of academic boredom (Zhou et al., 2017b, 2018).

Mobile phone dependence may play a mediating role in the relation between PTSD and academic boredom in adolescents. The model of compensatory internet use (Kardefelt-Winther, 2014) assumes that when adolescents encounter difficulties in the process of growing up, they readily engage in “pathological compensation” to meet their own development needs, such as dependence on the Internet (Gao and Chen, 2006). With the rapid development of information technology, mobile phones have become the main means by which to surf the Internet (China Internet Network Information Center, 2020). Given the various functions offered by mobile phone technology, mobile phones have gradually become the most common tool with which adolescents search the Internet (Liu et al., 2020). Adolescents with PTSD might use mobile phones to relieve their pressure and escape the dilemma (Contractor et al., 2018; Yu and Wang, 2020). In addition, these adolescents might have interpersonal communication difficulties in the real world, and therefore resort to the online world to meet their interpersonal needs (Contractor et al., 2018), thus forming an excessive dependence on mobile phones. Adolescents with a tendency to rely on their mobile phone spend a lot of time using it, which directly leads to a reduction in time invested in learning activities (Wentworth and Middleton, 2014). Furthermore, mobile phone dependence can lead to a decrease in attention, which makes it difficult for adolescents to invest the cognitive effort necessary for learning activities, and negatively affects their persistence and motivation (May et al., 2015; Zhen et al., 2020). All of these factors can lead to lower academic performance, which can result in a sense of academic boredom (May et al., 2015). Therefore, mobile phone dependence may mediate the relation between PTSD and academic boredom in adolescents.

**FIGURE 1:**
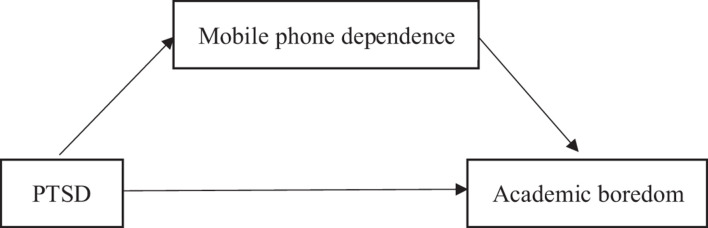
The hypothesized mediating effect model.

Few studies have explored the mechanism underlying the relation between PTSD and academic boredom in adolescents in the context of the COVID-19 pandemic. Accordingly, the present study recruited middle school students who had experienced the COVID-19 pandemic to test and expand previous related theories, and simultaneously investigate the reasons underlying academic boredom in adolescents during the pandemic, with the aim of providing theoretical evidence that can be used to alleviate their academic problems. On the basis of the job demands–resources model (Schaufeli and Bakker, 2004) and the model of compensatory internet use (Kardefelt-Winther, 2014), the following hypotheses were proposed: (1) PTSD would directly affect academic boredom in adolescents during the pandemic; (2) PTSD would indirectly affect academic boredom in adolescents through mobile phone dependence during the pandemic. The hypothetical intermediary model is shown in Figure 1 below.

## Materials and Methods

### Participants

Questionnaires were distributed to a senior high school in Hubei Province, China. The original sample consisted of 683 adolescents, but 54 of them were excluded as more than half of their data were missing on the variables we analyzed. A total of 631 valid questionnaires were finally obtained after collecting and sorting. The average age of participants was 16.06 years (*SD* = 0.56), ranging from 15 to 18 years; 336 were boys (53.2%) and 295 were girls (46.8%).

### Measures

#### Post-traumatic Stress Disorder

This study used a Chinese version (Zhou et al., 2017a) of the PTSD checklist for DSM-5 (Weathers, 2013) to assess adolescents’ PTSD symptoms relating to the COVID-19 pandemic. The checklist comprises 20 items and four subscales: intrusion symptoms (e.g., “Repeated, disturbing dreams of the pandemic”), avoidance symptoms (e.g., “Avoiding memories, thoughts, or feelings related to the pandemic”), negative alterations in cognition and mood (e.g., “Loss of interest in activities that I used to enjoy”), and hyperarousal symptoms (e.g., “Having difficulty concentrating”). Each item was scored on a 5-point scale ranging from 0 (not at all/only once) to 4 (almost every day). The summed scores of all items were taken as an indicator of frequency and severity of PTSD symptoms, whereby a higher score indicates more severe PTSD symptoms. In this study, the Cronbach’s alpha coefficient for the adapted scale was 0.89.

#### Academic Boredom

We used the boredom subscale of Academic Emotions Questionnaire developed by Dong and Yu (2007) to assess adolescents’ academic boredom (e.g., “I think learning is boring”). The subscale comprises 11 items, each of which is scored on a 5-point scale ranging from 1 (not at all) to 5 (completely agree). A higher summed score indicates a higher level of academic boredom. In this study, the Cronbach’s alpha coefficient for the subscale was 0.94.

#### Mobile Phone Dependence

The mobile phone dependence questionnaire developed by Seo et al. (2016). The questionnaire comprises 7 items (e.g., “The amount of time I spend using my mobile phone is increasing”), each of which is scored on a 4-point scale ranging from 1 (not at all) to 4 (completely agree). A higher summed score indicates more severe mobile phone dependence. In this study, the Cronbach’s alpha coefficient for the questionnaire was 0.85.

#### Procedure and Data Analysis

In July, 2020, about 6 months after the pandemic outbreak, under the help of a psychology teacher, we recruited grade one students from a senior high school in Hubei province, China to participate in our investigation. Since Wuhan is the first city to break out of COVID-19, it had been severely threatened by the COVID-19 pandemic, and the diagnosed COVID-19 cases in Wuhan ranked first among a total of 17 cities and districts in Hubei Province. Furthermore, Wuhan has experienced a lockdown, which will bring a negative impact on adolescents. Therefore, we take adolescents from Wuhan as our participates. This investigation was approved by the Research Ethics Committee of the Department of Psychological and Behavioral Sciences, Zhejiang University, and students provided their written informed consent to participate in this investigation. Before answering the questionnaire, the examiner explained the instructions and told participants that they are free to withdrawal at any time. Besides, we declared to them that the obtained data would be strictly confidential and would only be used for scientific research. Participants completed the questionnaire package within 30 min, and all questionnaires were collected on the spot.

SPSS19.0 was used for the descriptive analysis and correlation analysis, and Mplus7.0 was used for establishing mediating effect model. We also adopted bootstrap analysis (Preacher and Hayes, 2008) to examine the significance of the mediating effect. The significance was tested on the basis of whether the 95% confidence interval included 0 (Taylor et al., 2008). If not, this suggested that the indirect path was significant.

## Results

### Descriptive Statistics and Correlations Between Main Measures

Table 1 shows the descriptive statistics and correlations between PTSD, mobile phone dependence, and academic boredom scores. Gender and age were also included in the correlation analysis. As seen in Table 1, gender was significantly correlated with PTSD, but there were no significant correlations of gender with mobile phone dependence or academic boredom scores; there were no significant correlations of age with PTSD, mobile phone dependence, or academic boredom scores. Significant correlations were found between PTSD, mobile phone dependence, and academic boredom scores.

**TABLE 1:** Means, standard deviations, and correlations among main variables.

Main variables	M ± SD	1	2	3	4
1. Gender	–	–			
2. Age	16.06 ± 0.56	−0.13***	–		
3. PTSD	1.28 ± 0.68	0.09*	0.03	–	
4. Mobile phone dependence	2.56 ± 0.68	0.02	−0.00	0.36***	–
5. Academic boredom	2.53 ± 0.94	−0.02	−0.02	0.37***	0.46***

*P < 0.05, ***P < 0.001.

### The Mediating Role of Mobile Phone Dependence

First, a direct model was built to assess the direct effect of PTSD on academic boredom (controlling for gender). The direct model fitted the data well, χ^2^(0) = 0.000, RMSEA (90%CI) = 0.000, CFI = 1.000, TLI = 1.000, and SRMR = 0.000. The path analysis result indicated that PTSD was positively associated with academic boredom (β = 0.38, *p* < 0.01).

**FIGURE 2:**
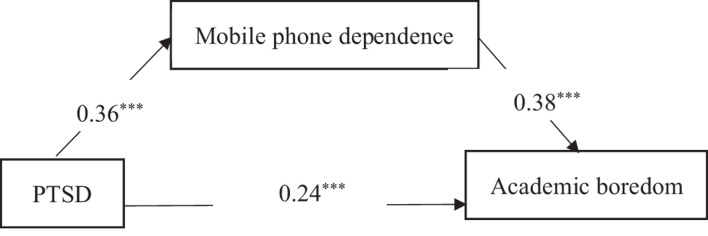
The mediating effect model of PTSD on academic boredom. ***P < 0.001.

Second, we established a mediating effect model to reveal the relations between PTSD, mobile phone dependence, and academic boredom. Regarding PTSD as a predictor variable and mobile phone dependence as a mediating variable (controlling for gender), this model showed a good fit with the data, χ^2^(0) = 0.000, RMSEA (90%CI) = 0.000, CFI = 1.000, TLI = 1.000, and SRMR = 0.000. As can be seen in Figure 2, all path coefficients were positive and significant. That is, PTSD was positively associated with academic boredom both in a direct way and in an indirect way via mobile phone dependence.

Finally, we examined the significance of the mediating effects above using a bootstrap analysis. Table 2 shows the bootstrap test results. The 95% confidence interval of the indirect paths from PTSD to academic boredom scores did not include 0, which indicates that the above-mentioned mediation effect was significant. The mediation effect value was 0.36 ^∗^ 0.38 = 0.14; that is, the mediation effect accounted for 14% of the total effect value.

**TABLE 2:** Bootstrap test of mediating effects.

Paths	95% CI	β
PTSD—Academic boredom	[0.16, 0.32]	0.24
PTSD—Mobile phone dependence	[0.29, 0.44]	0.36
Mobile phone dependence—Academic boredom	[0.30, 0.45]	0.38
PTSD—Mobile phone dependence—Academic boredom	[0.10, 0.18]	0.14

*Bootstrap sample size = 2000.*

## Discussion

This study explored the mediating role of mobile phone dependence in the relation between PTSD and academic boredom, so as to reveal the mechanism underlying the effect of PTSD on academic boredom under the COVID-19 pandemic.

### The Relation Between Post-traumatic Stress Disorder and Academic Boredom in Adolescents

We found that PTSD had a direct effect on academic boredom in adolescents, which supports hypothesis 1. Specifically, more severe PTSD symptoms were associated with a greater sense of boredom with adolescents’ learning, which is consistent with previous research on the relation between PTSD and academic boredom (Lin et al., 2013). One possible explanation for this result is that adolescents with PTSD require more individual resources (cognitive and emotional resources) to alleviate the negative outcomes caused by PTSD, which results in a lack of sufficient resources to deal with challenging learning tasks, especially when they study online alone during home quarantine under the pandemic. The mismatch between individual resources and needs can easily lead to academic boredom (Zhou et al., 2017b). In addition, adolescents with PTSD experience more problems in their daily lives, such as sleep disorders including insomnia and nightmares (Maguire et al., 2020). If adolescents do not get enough rest at night, they might find it difficult to deal with academic problems during the day; indeed, previous research has found that insufficient sleep causes adolescents to feel more pressure during the day (Maskevich et al., 2020). This pressure can lead to severe academic boredom, especially in adolescents who face the pressure of entering school (Lee et al., 2013).

### The Mediating Role of Mobile Phone Dependence

We found that PTSD not only had direct positive association with academic boredom in adolescents but also indirect positive association with academic boredom mediated by mobile phone dependence, which supports hypothesis 2. Specifically, the presence of more severe PTSD symptoms was associated with a stronger dependence on mobile phones and a greater sense of academic boredom. This supports previous findings of a positive correlation between PTSD and mobile phone dependence (Contractor et al., 2017). One possible reason for this association is that adolescents with PTSD tended to use mobile phones to constantly search for information related to the pandemic and watch pandemic-related videos, so as to gain the understanding of the pandemic and alleviate the unknown fear of the pandemic, and these behaviors make them fall into more serious mobile phone use (Elhai et al., 2020; Hu et al., 2021). In addition, these adolescents with PTSD might resort to use mobile phones to escape the pressures of society, school, and family (Yu and Wang, 2020), and regulate their emotions to increase positive emotions and reduce negative emotions, and maintain a balanced emotional state (Contractor et al., 2018). For example, entertainment and relaxation activities (e.g., music and games) provided by mobile phones can serve to reduce negative emotions (Jiang et al., 2016) and to deal with pain (Billieux et al., 2015). Besides, adolescents with PTSD are more sensitive to others’ behaviors and feelings (Slanbekova et al., 2019). Thus, face-to-face communication with others may bring additional pressures to them. This may encourage a reliance on mobile phones as a form of escape and compensation for their interpersonal communication in the real world (Zhen et al., 2019).

Excessive dependence on mobile phones can lead to academic boredom (Zhen et al., 2020). This may be because the excessive use of mobile phones not only means that adolescents spend less time studying (Samaha and Hawi, 2016), and adversely affect adolescents’ memory by diminishing chances for them to recollect things without the assistance of a mobile phone (Lewis et al., 2020), thus resulting in a decline of academic performance, but also that their attention is taken away from learning, the mobile phone’s determining characteristics (portable, swift, and accessible) may further make adolescents difficult to put down their mobile phones (Kliestik et al., 2020). Mobile phones as a distractor occupy adolescences’ cognitive resources, leading to poor performance (Thornton et al., 2014), which reduces their intrinsic learning motivations and leads to procrastination (Adams et al., 2020; Zhen et al., 2020). In addition, excessive use of mobile phones will breed negative emotions such as depression (Lazaroiu et al., 2020), which will also reduce their internal motivation (Elmelid et al., 2015). Furthermore, overuse in mobile phones may postpone adolescents’ bedtime routine, and night-time use activates ruminative thoughts and emotional involvement before sleep (Liu et al., 2017), causing shorter sleep duration and poorer sleep quality (Hong et al., 2020), which further leads to deficits in daily attention and memory (Ahmad and Bashir, 2017; Hong et al., 2020). These all do harm to adolescents’ performance in academic tasks, and breeds their academic boredom (Pekrun et al., 2017; Palos et al., 2019).

### Significance and Limitations

In summary, the present results have implications for educational practice; they help us to understand the factors and underlying mechanisms that affect adolescents’ academic boredom, and could help educators to reduce students’ academic boredom in the context of the COVID-19 pandemic. We found that PTSD impacts students’ academic boredom. Thus, teachers and parents should clarify the causes of academic boredom and acknowledge the impact of the pandemic on adolescents to be in a better position to help prevent the impact of the pandemic on their learning. Furthermore, we found that mobile phone dependence plays a mediating role in the relation between PTSD and academic boredom, which indicates that it is necessary to design and implement appropriate interventions to reduce mobile phone dependence among adolescents with PTSD.

This study has some limitations that should be acknowledged. First, this was a cross-sectional study, which makes it difficult to assess the changes in academic boredom of adolescents with PTSD under the pandemic over time. Second, this study used self-report questionnaires to evaluate the variables, and the deviation of the report may have affected the final results. Future longitudinal research should explore the current findings in more detail, while adopting diversified measurement methods to further enhance the reliability of the results.

## Conclusion

This study found that PTSD had a direct and positive relation with academic boredom in adolescents in the context of the COVID-19 pandemic, and mobile phone dependence mediated this relation. In addition, more severe PTSD symptoms were associated with a greater dependency on mobile phones and a greater sense of boredom with studies. To effectively address this problem in adolescents, teachers and parents should be aware of the impact of PTSD under the pandemic on adolescents and paid more attention to the problem of mobile phone dependence. From a theoretical perspective, the findings contribute to the extension of the job demands–resources model and the model of compensatory internet use theory from the standpoint of adolescents’ mental health. From an intervention perspective, our findings suggest that effective measures should be taken in schools and in the family environment to reduce adolescents’ dependence on mobile phones, and thus reduce their experienced boredom in learning activities.

## Data Availability Statement

The original contributions presented in the study are included in the article/supplementary material, further inquiries can be directed to the corresponding author/s.

## Ethics Statement

This investigation was approved by the Research Ethics Committee of the Department of Psychological and Behavioral Sciences, Zhejiang University, and students provided their written informed consent to participate in this investigation.

## Author Contributions

LS, XW, RZ, and XZ collected the data. LS and XW proofread the manuscript. RZ designed the study and performed data analysis. XZ performed data analysis and proofread the manuscript. All authors read and approved the manuscript.

## Conflict of Interest

The authors declare that the research was conducted in the absence of any commercial or financial relationships that could be construed as a potential conflict of interest.

## Publisher’s Note

All claims expressed in this article are solely those of the authors and do not necessarily represent those of their affiliated organizations, or those of the publisher, the editors and the reviewers. Any product that may be evaluated in this article, or claim that may be made by its manufacturer, is not guaranteed or endorsed by the publisher.

## References

[B1] Adams C. Grecu I. Grecu G. Balica R. (2020). Technology-related behaviors and attitudes: compulsive smartphone usage, stress, and social anxiety. *Rev. Contemp. Philos.* 19 71–77. 10.22381/RCP1920207

[B2] Ahmad S. Bashir S. (2017). A pilot study investigating the association between sleep and cognitive function among adolescents. *Asian J. Psychiatry* 28 34–37. 10.1016/j.ajp.2017.03.020 28784392

[B3] Billieux J. Thorens G. Khazaal Y. Zullino D. Achab S. Linden M. V. D. (2015). Problematic involvement in online games: a cluster analytic approach. *Comput. Hum. Behav.* 43 242–250. 10.1016/j.chb.2014.10.055

[B4] Cadime I. Pinto A. M. Lima S. Rego S. Pereira J. Ribeiro I. (2016). Well-being and academic achievement in secondary school pupils: the unique effects of burnout and engagement. *J. Adolesc.* 53 169–179. 10.1016/j.adolescence.2016.10.003 27814494

[B5] China Internet Network Information Center (2020). *The 46th China Statistical Report on Internet Development.* Available online at: http://www.cac.gov.cn/2020-09/29/c_1602939918747816.htm (accessed April 2, 2021).

[B6] Contractor A. A. Weiss N. H. Elhai J. D. (2018). Examination of the relation between PTSD symptoms, smartphone feature uses, and problematic smartphone use. *Soc. Sci. Comput. Rev.* 37:089443931877074. 10.1177/0894439318770745

[B7] Contractor A. A. Weiss N. H. Tull M. T. Elhai J. D. (2017). PTSD’s relation with problematic smartphone use: mediating role of impulsivity. *Comput. Hum. Behav.* 75 177–183. 10.1016/j.chb.2017.05.018 PMC13099003 42023016

[B8] Dekel S. Peleg T. Solomon Z. (2013). The relationship of PTSD to negative cognitions: a 17-year longitudinal study. *Psychiatry* 76 241–255. 10.1521/psyc.2013.76.3.241 23965263

[B9] Dong Y. Yu G. L. (2007). The development and application of an academic emotions questionnaire. *J. Psychol.* 39 852–860. 10.1080/10.1177/0143034318810318

[B10] Elhai J. D. Yang H. B. McKay D. Asmundson G. J. G. (2020). COVID-19 anxiety symptoms associated with problematic smartphone use severity in Chinese adults. *J. Affect. Disord.* 274 576–582. 10.1016/j.jad.2020.05.080 32663990 PMC7251360

[B11] Elmelid A. Stickley A. Lindblad F. Schwab-Stone M. Henrich C. C. Ruchkin V. (2015). Depressive symptoms, anxiety and academic motivation in youth: do schools and families make a difference? *J. Adolesc.* 45 174–182. 10.1016/j.adolescence.2015.08.003 26476790

[B12] Gao W. B. Chen Z. Y. (2006). A study on psychopathology and psychotherapy of internet addiction. *Adv. Psychol. Sci.* 14 596–603. 10.3969/j.issn.1671-3710.2006.04.018

[B13] Hong W. Liu R. D. Ding Y. Sheng X. Zhen R. (2020). Mobile phone addiction and cognitive failures in daily life: the mediating roles of sleep duration and quality and the moderating role of trait self-regulation. *Addict. Behav.* 107:106383. 10.1016/j.addbeh.2020.106383 32200196

[B14] Hu T. Wang Y. Lin L. Tang W. J. (2021). The mediating role of daytime sleepiness between problematic smartphone use and post-traumatic symptoms in COVID-19 home-refined adolescents. *Child Youth Serv. Rev.* 126:106012. 10.1016/j.childyouth.2021.106012 33846662 PMC8028598

[B15] Imran N. Aamer I. Sharif M. I. Bodla Z. H. Naveed S. (2020). Psychological burden of quarantine in children and adolescents: a rapid systematic review and proposed solutions. *Pak. J. Med. Sci.* 36 1106–1116. 10.12669/pjms.36.5.3088 32704298 PMC7372688

[B16] Jiang J. Ricksonb D. Jiang C. (2016). The mechanism of music for reducing psychological stress: music preference as a mediator. *Arts Psychother.* 48 62–68. 10.1016/j.aip.2016.02.002

[B17] Kardefelt-Winther D. (2014). A conceptual and methodological critique of internet addiction research: towards a model of compensatory internet use. *Comput. Hum. Behav.* 31 351–354. 10.1016/j.chb.2013.10.059

[B18] Kliestik T. Scott J. Musa H. Suler P. (2020). Addictive smartphone behavior, anxiety symptom severity, and depressive stress. *Analysis Metaphys.* 19 45–51. 10.22381/AM1920204

[B19] Lazaroiu G. Kovacova M. Siekelova A. Vrbka J. (2020). Addictive behavior of problematic smartphone users: the relationship between depression, anxiety, and stress. *Rev. Contemp. Philos.* 19 50–56. 10.22381/RCP1920204

[B20] Lee J. Puig A. Lea E. Lee S. M. (2013). Age-related differences in academic burnout of korean adolescents. *Psychol. Sch.* 50 1015–1031. 10.1002/pits.21723

[B21] Lewis J. Pera A. Balica R. (2020). Problematic smartphone use severity, psychiatric symptoms, and behavioral addiction. *Analysis Metaphys.* 19 66–72. 10.22381/AM1920207

[B22] Lin C. D. Wu X. C. Zhang Y. D. Zang W. W. Zhou X. Dai Y. (2013). Investigation on mental health state of primary and secondary school students after 30 months of Wenchuan earthquake. *Psychol. Dev. Educ.* 29 631–640. 10.16187/j.cnki.issn1001-4918.2013.06.005

[B23] Lin S. H. Huang Y. C. (2014). Life stress and academic burnout. *Act. Learn. High. Educ.* 15 77–90. 10.1177/1469787413514651

[B24] Liu Q. Q. Yang X. J. Hu Y. T. Zhang C. Y. Nie Y. G. (2020). How and when is family dysfunction associated with adolescent mobile phone addiction? Testing a moderated mediation model. *Child Youth Serv. Rev.* 111:104827. 10.1016/j.childyouth.2020.104827

[B25] Liu Q. Q. Zhou Z. K. Yang X. J. Kong F. C. Niu G. F. Fan C. Y. (2017). Mobile phone addiction and sleep quality among Chinese adolescents: a moderated mediation model. *Comput. Hum. Behav.* 72 108–114. 10.1016/j.chb.2017.02.042

[B26] Maguire D. G. Ruddock M. W. Milanak M. E. Moore T. Armour C. (2020). Sleep, a governor of morbidity in ptsd: a systematic review of biological markers in PTSD-related sleep disturbances. *Nat. Sci. Sleep* 12 545–562. 10.2147/NSS.S260734 32801980 PMC7402856

[B27] Marchini S. Zaurino E. Bouziotis J. Brondino N. Delvenne V. Delhaye M. (2020). Study of resilience and loneliness in youth (18-25 years old) during the COVID-19 pandemic lockdown measures. *J. Commun. Psychol.* 49 468–480. 10.1002/jcop.22473 33169377

[B28] Maskevich S. Cassanet A. Allen N. B. Trinder J. Bei B. (2020). Sleep and stress in adolescents: the roles of pre-sleep arousal and coping during school and vacation. *Sleep Med.* 66 130–138. 10.1016/j.sleep.2019.10.006 31877504

[B29] May R. W. Bauer K. N. Fincham F. D. (2015). School burnout: diminished academic and cognitive performance. *Learn. Individ. Differ.* 42 126–131. 10.1016/j.lindif.2015.07.015

[B30] Palos R. Maricutoiu L. P. Costea I. (2019). Relations between academic performance, student engagement and student burnout: a cross-lagged analysis of a two-wave study. *Stud. Educ. Eval.* 60 199–204. 10.1016/j.stueduc.2019.01.005

[B31] Pekrun R. Lichtenfeld S. Marsh H. W. Murayama K. Goetz T. (2017). Achievement emotions and academic performance: longitudinal models of reciprocal effects. *Child Dev.* 88 1653–1670. 10.1111/cdev.12704 28176309

[B32] Perry J. C. Liu X. Pabian Y. (2010). School engagement as a mediator of academic performance among urban youth: the role of career preparation, parental career support, and teacher support. *Couns. Psychol.* 38 269–295. 10.1177/0011000009349272

[B33] Preacher K. J. Hayes A. F. (2008). Asymptotic and resampling strategies for assessing and comparing indirect effects in multiple mediator models. *Behav. Res. Methods* 40 879–891. 10.3758/BRM.40.3.879 18697684

[B34] Qi Y. J. Wu X. C. (2018). Job demands-resources model: the development of theoretical and empirical research. *J. Beijing Norm. Univ.* 270 30–38.

[B35] Quin D. (2016). Longitudinal and contextual associations between teacher-student relationships and student engagement. *Rev. Educ. Res.* 20 1–43. 10.3102/0034654316669434

[B36] Rojas-Flores L. Herrera S. Currier J. M. Foster J. D. Putman K. M. Roland A. (2015). Exposure to violence, posttraumatic stress, and burnout among teachers in el salvador: testing a mediational model. *Int. Persp. Psychol. Res. Pract. Cons.* 4 98–110. 10.1037/ipp0000029

[B37] Samaha M. Hawi N. S. (2016). Relationships among smartphone addiction, stress, academic performance, and satisfaction with life. *Comput. Hum. Behav.* 57 321–325. 10.1016/j.chb.2015.12.045

[B38] Schaufeli W. B. Bakker A. B. (2004). Job demands, job resources, and their relationship with burnout and engagement: a multi-sample study. *J. Organ. Behav.* 25 293–315. 10.1002/job.248

[B39] Seo D. G. Park Y. Kin M. K. Park J. (2016). Mobile phone dependency and its impacts on adolescents’ social and academic behaviors. *Comput. Hum. Behav.* 63 282–292. 10.1016/j.chb.2016.05.026

[B40] Slanbekova G. K. Chung M. C. Karipbaev B. I. Sabirova R. S. Alimbayeva R. T. (2019). Posttraumatic stress and interpersonal sensitivity: alexithymia as mediator and emotional expressivity as moderator. *Psychiat. Quart.* 90 1–13. 10.1007/s11126-018-9612-5 30515699

[B41] Supervía P. U. Bordás C. S. Lorente V. M. (2020). Psychological analysis among goal orientation, emotional intelligence and academic burnout in middle school students. *Int. J. Environ. Res. Public Health* 17:8160. 10.3390/ijerph17218160 33158255 PMC7662988

[B42] Taylor A. B. MacKinnon D. P. Tein J. Y. (2008). Tests of the three-path mediated effect. *Organ. Res. Methods* 11 241–269. 10.1177/1094428107300344

[B43] Thornton B. Faires A. Robbins M. Roiilins E. (2014). The mere presence of a cell phone may be distracting. *Soc. Psychol.* 45 479–488. 10.1027/1864-9335/a000216

[B44] Weathers F. W. (2013). “The PTSD checklist for DSM-5(PCL-5): development and initial psychometric analysis”, in *Paper Presented at the 29th Annual Meeting of the International Society for Traumatic Stress Studies* (Philadelphia, PA: ISTSS).

[B45] Wentworth D. K. Middleton J. H. (2014). Technology use and academic performance. *Comput. Educ.* 78 306–311. 10.1016/j.compedu.2014.06.012

[B46] Wiguna T. Anindyajati G. Kaligis F. Ismail R. I. Pradana K. (2020). Brief research report on adolescent mental well-being and school closures during the COVID-19 pandemic in indonesia. *Front. Psychiatry* 11:598756. 10.3389/fpsyt.2020.598756 33312144 PMC7704451

[B47] Xu W. An Y. Y. Ding X. Yuan G. Z. Zhuang Y. L. Goh P. H. (2017). Dispositional mindfulness, negative posttraumatic beliefs, and academic burnout among adolescents following the 2016 Yancheng tornado. *Pers. Indiv. Differ.* 116 405–409. 10.1016/j.paid.2017.05.029

[B48] Ying L. H. Wang Y. L. Lin C. D. Chen C. S. (2016). Trait resilience moderated the relationships between PTG and adolescent academic burnout in a post-disaster context. *Pers. Indiv. Differ.* 90 108–112. 10.1016/j.paid.2015.10.048

[B49] Yu G. L. Wang P. C. (2020). Psychological effect and response of cell phone dependence - A case study of COVID-19. *J. Soc. Sci.* 5 58–66.

[B50] Yue J. M. Zang X. Y. Le Y. Y. An Y. Y. (2020). Anxiety, depression and PTSD among children and their parent during 2019 novel coronavirus disease (COVID-19) outbreak in China. *Curr. Psychol.* 1 1–8. 10.1007/s12144-020-01191-4 33223783 PMC7666617

[B51] Zhang C. Y. Ye M. L. Fu Y. W. Yang M. Y. Luo F. Yuan J. H. (2020). The psychological impact of the COVID-19 pandemic on teenagers in China. *J. Adolesc. Health* 67 747–755. 10.1016/j.jadohealth.2020.08.026 33041204 PMC7543885

[B52] Zhen R. Li L. Ding Y. Hong W. Liu R. D. (2020). How does mobile phone dependency impair academic engagement among chinese left-behind children? *Child. Youth Serv. Rev.* 116:105169. 10.1016/j.childyouth.2020.105169

[B53] Zhen R. Liu R. D. Hong W. Zhou X. (2019). How do interpersonal relationships relieve adolescents’ problematic mobile phone use? The roles of loneliness and motivation to use mobile phones. *Int. J. Environ. Res. Public Health* 16:2286. 10.3390/ijerph16132286 31261619 PMC6650804

[B54] Zhen R. Zhou X. (2021). Latent patterns of posttraumatic ptress pymptoms, depression, and posttraumatic growth among adolescents during the COVID-19 pandemic. *J. Trauma Stress* 16:2286. 10.1002/jts.22720 PMC8426724 34339577

[B55] Zhou X. Zhen R. Wu X. C. (2017b). Posttraumatic stress disorder symptom severity and control beliefs as the predictors of academic burnout amongst adolescents following the Wenchuan earthquake. *Eur. J. Psychotraumatol.* 8:1412227. 10.1080/20008198.2017.1412227 29296242 PMC5738653

[B56] Zhou X. Wu X. Zhen R. (2017a). Assessing the latent structure of DSM-5 PTSD among Chinese adolescents after the Ya’an earthquake. *Psychiatry Res.* 254 33–39. 10.1016/j.psychres.2017.04.029 28441585

[B57] Zhou X. Wu X. C. An Y. Y. Fu F. (2014). Longitudinal relationships between posttraumatic stress symptoms and sleep problems in adolescent survivors following the Wenchuan earthquake in China. *PLoS One* 9:e104470. 10.1371/journal.pone.0104470 25105288 PMC4126730

[B58] Zhou X. Zhen R. Wu X. C. (2018). Trajectories of academic burnout in adolescents after the Wenchuan earthquake: a latent growth mixture model analysis. *Sch. Psychol. Int.* 40 183–199. 10.1177/0143034318810318

